# The impact of heat stress in plant reproduction

**DOI:** 10.3389/fpls.2023.1271644

**Published:** 2023-12-07

**Authors:** Francesca Resentini, Gregorio Orozco-Arroyo, Mara Cucinotta, Marta A. Mendes

**Affiliations:** Dipartimento di Bioscienze, Università degli Studi di Milano, Milano, Italy

**Keywords:** heat stress, plant reproduction, rice, *Arabidopsis*, calcium signaling, pollen development, ovule development

## Abstract

The increment in global temperature reduces crop productivity, which in turn threatens food security. Currently, most of our food supply is produced by plants and the human population is estimated to reach 9 billion by 2050. Gaining insights into how plants navigate heat stress in their reproductive phase is essential for effectively overseeing the future of agricultural productivity. The reproductive success of numerous plant species can be jeopardized by just one exceptionally hot day. While the effects of heat stress on seedlings germination and root development have been extensively investigated, studies on reproduction are limited. The intricate processes of gamete development and fertilization unfold within a brief timeframe, largely concealed within the flower. Nonetheless, heat stress is known to have important effects on reproduction. Considering that heat stress typically affects both male and female reproductive structures concurrently, it remains crucial to identify cultivars with thermotolerance. In such cultivars, ovules and pollen can successfully undergo development despite the challenges posed by heat stress, enabling the completion of the fertilization process and resulting in a robust seed yield. Hereby, we review the current understanding of the molecular mechanisms underlying plant resistance to abiotic heat stress, focusing on the reproductive process in the model systems of *Arabidopsis and Oryza sativa.*

## Introduction

Heat Stress (HS) causes substantial crop loss worldwide. The average global temperature is constantly increasing, and this change is expected to have deleterious effects on crop yield. A recent study showed that drought and, particularly, extreme heat episodes dramatically decreased cereal production by 9–10% between 1964 and 2007 (Lesk et al., 2016). Average temperatures are estimated to rise by 2–3°C over the next 30 to 50 years. Given the fact that the human population is estimated to reach 9 billion by 2050, genetic improvement of tolerance traits to abiotic stresses on stable crops is an immediate priority. Europe recently experienced several heat waves, in 2003 the heat-related death toll ran into tens of thousands. Another heat wave in 2012 impacted crop productivity and yield of several important food species with a decrease of up to 40%, as for sunflowers for example (Peng et al., 2004). Among all the documented losses, it was estimated that rice grain production decreased by 10% for each 1°C increase, and it has also been predicted that every 1°C increase reduces wheat production by 3 - 4% (Xu et al., 2020; Wang et al., 2020). Similar deleterious effects have been shown for maize and barley, for which each day that the plants are exposed to a temperature over 30°C, yield is reduced by 1% (Rezaei et al., 2015). The year 2016 ranks as the warmest on record and the year 2018 was the fourth warmest since 1880 (Source: NASA/GISS) confirming a continuous trend towards warmer climates. Since extreme climatic events, such as heat waves, are increasingly common, agriculture will face extraordinary challenges to sustain productivity (Mulla et al., 2020).

**Figure 1:**
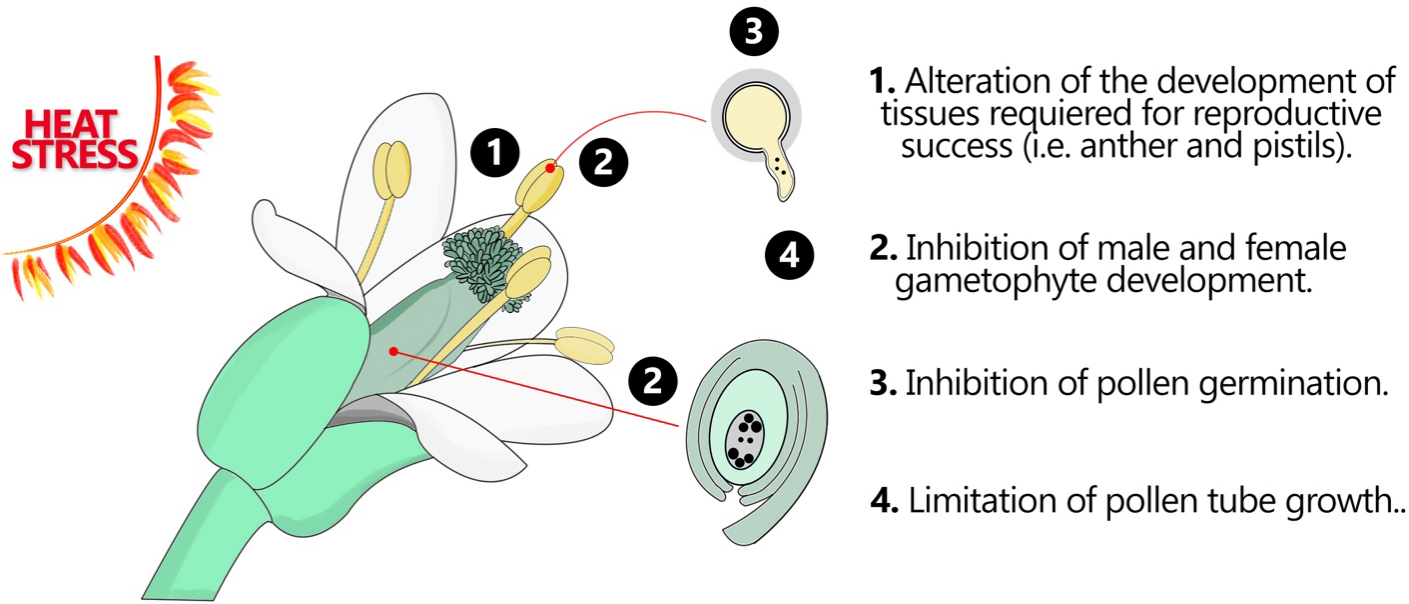
Flower organs and tissues in response HS. Heat stress can have significant impacts on various flower organs and tissues, leading to alterations in their development, structure, and function. These effects can disrupt normal floral development and ultimately impact reproductive success. This figure demonstrates how the fertile flower organs may respond to HS.

Understanding how plants cope with HS during their reproductive phase is critical for managing the future of agricultural productivity, as most of our food supply is a product of plant reproduction. Even if the effect of temperature has been extensively studied using accessible plant tissues, such as leaves and roots, analysis on reproduction is often difficult because gametophyte development and fertilization are complex processes that occur during a narrow window of time and deep inside the flower. The effect of HS on plant reproduction is very wide affecting many reproductive tissues at the same time. HS leads to abnormalities in floral development, plants develop altered flower structures, with reduced flower size, or even the development of complete sterile flowers. These flower changes are translated into impaired pollination and fertilization processes that ultimately lead to reduced fruit and seed production. From the male side, HS impacts anther and pollen grain development leading to morphological abnormalities and displacement of the metabolic processes that impair pollen grain ability to germinate and to grow pollen tubes. From the female side, HS can disrupt gametogenesis, leading to abnormal development of the female gametophyte (embryo sac), this can affect the formation of essential components within the embryo sac, such as the gametes (egg cell). Impaired fertilization and embryo development are the ultimate consequences of HS which results in reduced and/or poor seed production (Barnabás et al., 2008; Prasad et al., 2008; Bita and Gerats, 2013) (**Figure 1**).

Many genes regulating responses and resistance to various biotic and abiotic stresses have been precisely identified. However, coordinated responses between roots and leaves at the whole plant level remain largely unknown. Roots and shoots communicate with each other to synchronize and optimize plant development and respond to environmental changes. Thus, the growth of these two structures is coordinated and this requires communication mediated by signal messengers that move between the aboveground and belowground structures (Ko and Helariutta, 2017). The vascular system serves as an effective long‐distance communication system, with the phloem and xylem serving to input information relating to conditions (Lucas et al., 2013). An interesting observation is that a stress applied to systemic tissues is sensed by the inflorescence in a short timeframe (minutes or a few hours) (Choudhury et al., 2018), thus pointing to the existence of a fast communication pathway that most probably relies on (i) changes in electric potential, (ii) calcium ions (Ca^2+^) and (iii) reactive oxygen species (ROS) (Iba, 2002; Liao et al., 2017; Jespersen, 2020).

For this reason, understanding how environmental cues are sensed and transmitted to systemic organs of plants such as the inflorescence and how this impacts the flower development therefore seeds’ setting and plant reproduction in model species, such as *Arabidopsis*, could be relevant for the establishment of a baseline for crop improvement. Here we will provide an overview of the importance of molecular mechanisms underlying plant resistance to HS, focusing on the reproductive process in *Arabidopsis thaliana* and *Oryza sativa*, highlighting the role of Ca^2+^ as a link between the perception of environmental signal and a physiological response.

## Pollen and ovule development response to HS in Arabidopsis

Flowering plants, i.e. angiosperms, alternate between a highly reduced gametophytic (haploid) and sporophytic (diploid) generations (Yadegari and Drews, 2004). The sporophyte is the multicellular diploid plant whereas the haploid structure called gametophyte is generated by meiotic cell division within the male and female reproductive organs (Drews and Koltunow, 2011). In Arabidopsis, male gametophyte development occurs within stamens, composed of a filament and an anther. Within anthers, non-reproductive cells differentiate into specialized layers, including the tapetum, surrounding sporogenous cells (Scott et al., 2004). Two distinct phases, microsporogenesis and microgametogenesis, produce mature pollen. Microsporogenesis involves meiosis in pollen mother cells, generating haploid microspores. After callose wall degeneration, individual microspores are released (Borg et al., 2009). Subsequent mitotic divisions yield vegetative and generative cells. Asymmetric division in the first pollen mitosis determines unique gene expression profiles, defining structures and fates. A second mitosis produces twin sperm cells for double fertilization, leading to embryo and endosperm development (Twell et al., 1998). Abiotic stress in pollen development was comprehensively studied in the last years and reviews were produced where pollen defects in different species were reviewed (Chaturvedi et al., 2021).

**Figure 2:**
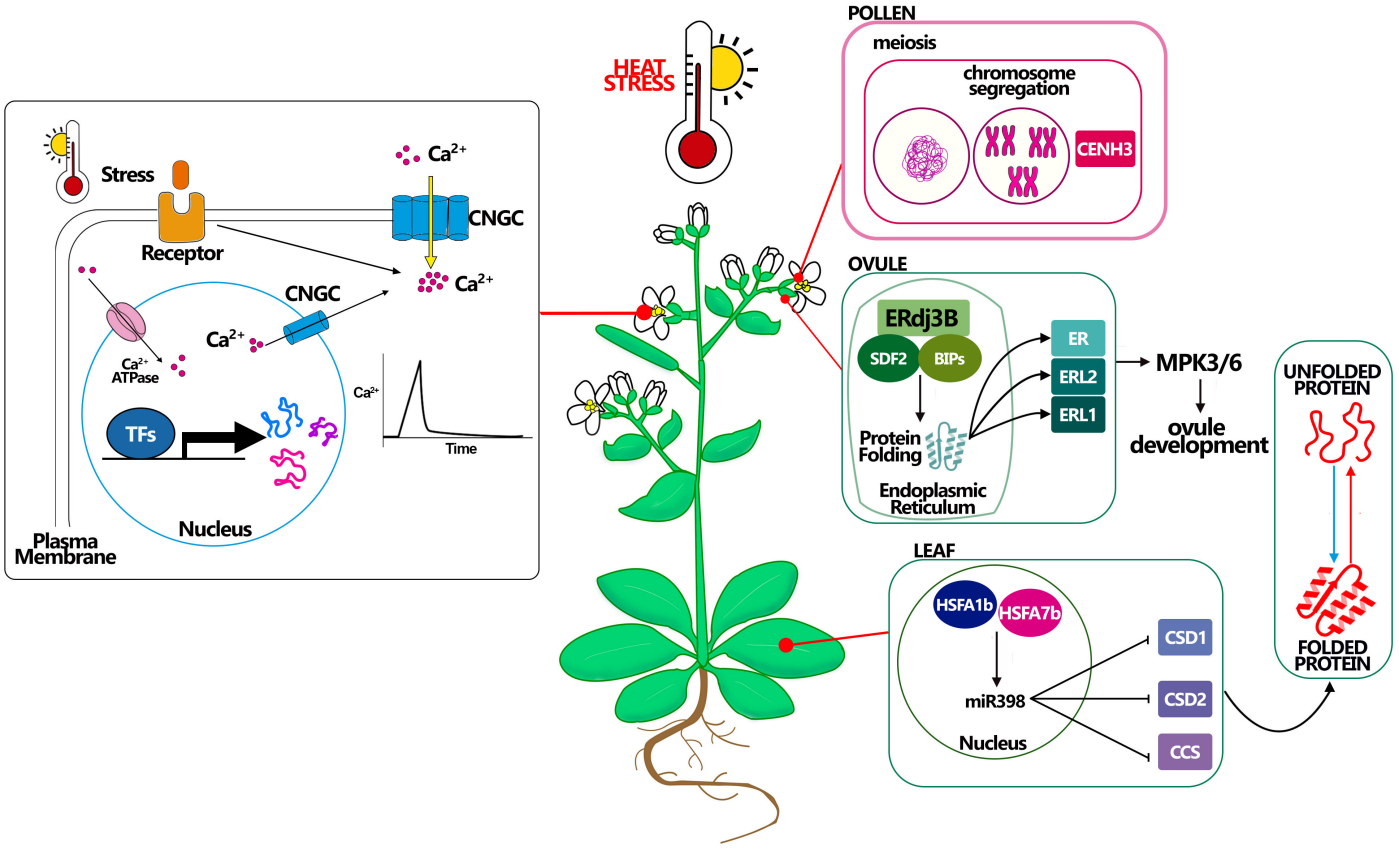
Molecular mechanisms connected with heat stress resistance in Arabidopsis. Heat affects plant Ca^2+^ channels, inducing a transient increase in cytosolic Ca^2+^ concentration. Stress sensors recognize environmental signals (i.e. heat) and activate plasma membrane-localized Ca^2+^ channels, allowing Ca^2+^ influx into the cytosol by the use of CNGCs channels. Cytosolic Ca^2+^ ion acts as a second messenger that triggers specific cellular responses. Pollen CENH3 mediates genome elimination during HS, this mechanism could be better studied to understand how to maintain meiosis under HS. Ovule complex ERdj3B acting in complex with SDF2 and BIPs in the endoplasmic reticulum control the protein folding, being essential to the plant tolerate the HS, probably via the ERL proteins. A good example in leaf development for thermotolerance is giving by miR398 which expression is controlled by HSFA proteins and that promotes the resistance to stress controlling the correct folding of the proteins.

In Arabidopsis was recently demonstrated that the male gametophyte (pollen) is particularly sensitive to heat fluctuations, causing defects in meiotic restitution (De Storme and Geelen, 2020). More in detail meiosis and in particular meiotic recombination are highly sensitive to elevated temperatures, meiotic microtubule cytoskeleton resulted in an irregular spindle orientation, and aberrant cytokinesis that consequently led to the production of aneuploid male gametes (De Storme and Geelen, 2020; Hedhly et al., 2020; De Jaeger-Braet et al., 2022). In Arabidopsis, the increase in crossover frequency at high temperatures was associated with elevated numbers of Type I interfering pathway crossover. Interestingly, the meiotic hyper-recombination observed in Arabidopsis resulted specific for HS, as plants subject to salt stress did not exhibit an increase in crossover frequency (Modliszewski et al., 2018). Precisely because of the HS effect on chromosome segregation, high-temperature treatment has been proposed as a tool in plant breeding to induce genome elimination and haploid induction. Indeed, if applied to haploid inducer mutants, such as mutant for the CENTROMERE-SPECIFIC HISTONE H3, short-term HS increases the efficiency of haploid induction by ten times (Ahmadli et al., 2023; Jin et al., 2023; **Figure 2**). Another aspect of male meiosis that is influenced by the HS is the duration of the different phases of meiosis. By performing live cell imaging on male meiocytes, De Jaeger-Braet et al. (2022) showed that the meiosis phase of meiocytes at a high temperature of 34°C is faster than at 21°C. By contrast, the pachytene/diakinesis phase gets prolonged at 34°C. The extension of this specific phase is recombination dependent since it was not detected in *ataxia telangiectasia mutated* (*atm*) mutant in which recombination is completely abolished.

Regarding the female part of development studies using the model plant Arabidopsis, HS reduced the total number of ovules and increased ovule abortion (Whittle et al., 2009). Furthermore, it has been described in Tomato that female pistils exposed to HS (32/26°C) and then crossed with pollen from plants grown in standard conditions (at 28/22°C) exhibited reduced fruit set and a reduced number of seeds per fruit compared with the control pistils of plants grown at 28/22°C (Peet et al., 1998). The seed set was even more reduced in the reciprocal experiment using maize plants when transient HS was applied for three days on developing pollen that later was used to pollinate female flowers grown at optimal conditions (Begcy et al., 2019). These were the only studies that described some of the female defects caused by HS, which remained largely unstudied.

In Arabidopsis, the female germline initiates during the initial phase of ovule development. This process begins with the differentiation of a distal subepidermal cell known as the megaspore mother cell, which undergoes meiosis to give rise to four haploid megaspores, typically arranged in a linear tetrad. Among these four megaspores, only one survives to become the functional megaspore, while the remaining three undergo programmed cell death. Following megasporogenesis, the functional megaspore proceeds to undergo a Polygonum-type pattern of megagametogenesis, leading to the formation of the embryo sac and female gamete. Integument development in Arabidopsis is a simple two-cell layered structure that develops around the embryo sac and after fertilization protects the developing embryo developing the seed coat (Christensen et al., 2002; Yadegari and Drews, 2004, Mendes et al., 2016).

Arabidopsis endoplasmic reticulum-localized DnaJ family 3B (ERdj3B) was recently described as an important factor for the correct development of ovule integuments by controlling the translocation of the ERECTA-family receptor kinases in the ecotype Landsberg (Leng et al., 2022). ERdj3B is a component of the stromal cell-derived factor 2 (SDF2)–ERdj3B–binding immunoglobulin protein (BiP) chaperone complex, and has functions in protein folding, translocation, and quality control. ERdj3B was first described to be involved in thermotolerance during anther development (Yamamoto et al., 2020) and was also described now to have complex functions not only in ovule integument development but also in HS response from the female side. Leng and collaborators (Leng et al., 2022) further described that higher temperatures were shown to aggravate the defective phenotypes of erdj3b mutants, linking that the response to HS has more severe effects on ovule development when ERECTA-family receptor kinases are absent (**Figure 2**). A recent study also described the TCP transcription factors inhibit the homeotic conversion of ovules into carpelloid structures under HS, (Lan et al., 2023) reinforcing the fact that transcription factor complexes important for ovule identity can also be related to HS perception and could have significant implications for understanding the molecular mechanisms underlying the plant’s response to HS and how it affects reproductive development.

## HS proteins and HS transcription factors in Arabidopsis

For quite some time in cellular biology, HS proteins have been recognized as those whose levels significantly rise when cells are cultivated at elevated temperatures, providing a form of resilience. It is now understood that these proteins play a role in assisting newly synthesized proteins in proper folding and safeguarding proteins that may otherwise misfold and lose their intended functional conformation during stressful events. Importantly, these proteins are not solely associated with HS but also have connections to other biotic and/or abiotic stress conditions. HS proteins (HSPs) besides stress-responsive genes (Ul Haq et al., 2019), also are involved in plant growth and development under normal conditions, like the flowers, seeds, and fruits set development, in the tuberization (Agrawal et al., 2013) and nutrient uptake (Shekhar et al., 2016). Studies using Arabidopsis and crops (rice, maize, and wheat) showed that the basis of thermotolerance resides in the overexpression of HSP factors, which increase plant resistance to abiotic HS (Ul Haq et al., 2019). These studies have focused on the analysis of transgenic plants under a broad spectrum of induced HS treatments, which makes the data extremely variable and not suitable for comparison purposes (Yeh et al., 2012). The optimum scenario is to identify a stable accession or species that naturally overexpresses HSP factors that induce HS resistance. Furthermore, most of the thermotolerance studies are again based on seedling germination and root development, meanwhile, the reproductive phase is often not considered. Therefore, even though a plant can potentially tolerate HS at early phases of development, it might be HS susceptible at mature stages and hence sterile.

At the molecular level, the cellular response to HS is represented by the induction of HSP, a group of stress proteins that are classified as molecular chaperones and proteases. The molecular analysis of HSP promoters leads to the identification of the heat shock element (HSE), a stress-responsive promoter element essential for HS inducibility; this binding site is characterized by multiple adjacent 5`-nGAAn-3`. The position of HSEs in the genome is various and distances upstream of their transcription starting site. In vertebrates and plants, HSP transcription requires the transient binding of HS transcription factors (HSFs) to the HSEs present within their promoters (Wu, 1995; Morimoto, 1998; Kovács et al., 2022). Plant HSFs are divided in three classes A, B and C. Class A HSFs typically contain one or two acidic AHA motifs and function as transcriptional activators, as indicated by Döring et al. (2000). On the other hand, class B HSFs possess a B3 repressing domain, which has also been identified in 24 other transcription factors in Arabidopsis. Class C HSFs have not been thoroughly described (Czarnecka-Verner et al., 2004; Ikeda and Ohme-Takagi, 2009; Guo et al., 2016). In contrast to the limited number of HSF members in vertebrates (4), Drosophila (1), *Caenorhabditis elegans* (1), and yeast (1), plant HSF families exhibit a considerable number of members derived from a complex, plant-specific superfamily, as highlighted by Wu (1995) and Morimoto (1998).

The large size of the plant HSFs family inevitably complicates the unraveling of their function under stress conditions (Scharf et al., 2012 and reviewed in Guo et al., 2016).

The identification of factors that allow plants to tolerate HS has been mainly performed in the model species Arabidopsis. Yet, research on economically relevant species has been performed (Ul Haq et al., 2019). In Arabidopsis during the vegetative phase, the constitutive expression of the HS transcription factors HSFA1a, b, d, and e are responsible for triggering the HS response (Yoshida et al., 2011). *HSF1abde* are responsible for basal thermotolerance and initiate the acquisition of thermotolerance. A second transcription factor from this family is HSFA2, the most highly heat-induced HSF. Remarkably, ectopic expression of *HSFA2* was able to rescue the phenotype of the quadruple mutant *hsaf1abde* at reproductive stage (Liu and Charng, 2013). This is partly explained by the fact that HSFA2 can induce its own expression. HSFA3, HSFA7a, and HSFA7b are also induced by HSFA2 and/or HSFA1 after HS (Liu and Charng, 2013). Instead, the defective mutant *hsfa2* is only impaired in maintaining the acquired thermotolerance after long recovery (Charng et al., 2007). HSFs were extensively reviewed by Guo et al., 2016.

Manipulation on genes that have the potential to improve HS tolerance has focused mainly on genes involved in the synthesis of HSP. Yet, no clear evidence regarding the improvement to HS resistance of such mutants is available. One of the first studies that reported how a HSP manipulated can improve the tolerance to HS was made using the HSF SQUAMOSA promoter binding protein-like7 (SPL7, Yamanouchi et al., 2002). Mutant plants lacking the function of *spl7* develop more necrotic lesions on leaves under HS treatment (35°C for 24 hours followed by 42°C for 24 hours). *spl7* mutant lines complemented with wild-type SPL7 were more resistant to HS showing no occurrence of necrotic lesions during the growth period (Yamanouchi et al., 2002). Remarkably, a strong correlation between the inserted number of copies of the transgene and the reduction in the necrotic lesions was detected, suggesting that the “overexpression” of *SPL7* might be helpful to improving HS tolerance (Yamanouchi et al., 2002), any evidence in the reproductive part were studied. The sole HSF identified with a function in ovule development is HSFB2a. Plants with heterozygous mutations in HSFB2a display 50% sterile ovules and a significant decrease in both male and female transmission, suggesting that the gene’s absence adversely affects the development of both male and female germ lines. Even if is not an HS related phenotype is it very interesting to notice that the homozygous mutant was already sterile, with block during female gametophyte (Wunderlich et al., 2014). A very interesting study demonstrated that heat-inducible miR398 that is directly activated by HSFA1b and HSFA7b is required for thermotolerance through the downregulation of its target genes CSD1, CSD2 and CCS which encode for copper chaperones. The corresponding mutations to *csd1*, *csd2* and *ccs* mutant plants are more heat-tolerant and the resistant transgenic plants expressing the miR398-resistant forms of CSD1, CSD2 or CCS were more sensitive to HS at 37°C (Guan et al., 2013, **Figure 2**). Studies involving miRNAs during reproductive tissues would be of outmost importance as in the last years were described to play several roles during reproduction (Petrella et al., 2021).

## Calcium as a signal for HS in Arabidopsis

In plants, calcium ion (Ca^2+^) plays an important role both as a structural component of plant cell walls and membranes and as an intracellular second messenger. As second messenger, Ca^2+^ is involved in an advanced network of signaling pathways taking part in various signaling processes generated in response to both biotic and abiotic stresses, as well as developmental stimuli (Kudla et al., 2018; Resentini et al., 2021; Ghosh et al., 2022). In nature, plants must cope with both seasonal and diurnal temperature changes. Particularly, temperature fluctuations that occur in a single day can be dangerous for plants since they can face temperature stress more rapidly as compared to other stresses such as drought or salinity (Larkindale and Knight, 2002; Ghosh et al., 2022). Therefore, like other organisms, plants have evolved defense mechanisms to efficiently cope with temperature stress and to prevent the disruption of multiple cellular processes, including protein folding, cytoskeletal organization, membrane stability, regulation of ROS and ion homeostasis (Weigand et al., 2021; Ghosh et al., 2022).

It has been shown that plants exposed to a heat shock show a transient increase of the cytosolic Ca^2+^ concentration (Gong et al., 1998; Wu and Jinn, 2010). Such an increase was shown to depend on the activity of some members of the CYCLIC NUCLEOTIDE-GATED channels (CNGCs) family (Finka et al., 2012; Cui et al., 2020). Interestingly, CNGCs have been implicated in diverse aspects of plant growth and development, such as pollen tube growth and fertility. Six CNGC members—CNGC7, 8, 9, 10, 16 and 18—have been reported as highly expressed in the pollen grain and pollen tube (Frietsch et al., 2007; Tunc-Ozdemir et al., 2013). Among them, genetic evidence identifies CNGC16 as a critical component in maintaining pollen fertility under conditions of heat and drought stress (Tunc-Ozdemir et al., 2013). The *cngc16* mutant, in fact, showed more than a 10-fold stress-dependent loss in pollen fitness as well as seed set under HS and drought stress. At the same time, *cngc16* mutant pollen exhibited attenuated expression of HS responsive genes (Tunc-Ozdemir et al., 2013). Nonetheless, there are scant pieces of evidence directly supporting a role for Ca^2+^ signals as an initial heat sensing response during plant reproduction (Ghosh et al., 2022) (**Figure 2**).

Weigand and colleagues in 2021 generated a reporter called CGf, a ratiometric, genetically encoded Ca^2+^ indicator with a mCherry domain fused to the intensiometric Ca^2+^ reporter GCaMP6f. By using this new tool, the authors showed that HS suppressed the tip-focused Ca^2+^ oscillations in growing pollen tubes with the consequent growth arrest and even pollen tube tip rupture (Weigand et al., 2021). This important result highlights the urgent need to better investigate the HS signaling in pollen tubes and better define the role of Ca^2+^ signaling components in this response. It is obvious that the temperature stress, by affecting pollen tube development will lead to decreased fertility and reduced seed production. A good knowledge of the specific role of Ca^2+^ signaling in a pollen tube, subjected to HS, will surely be instrumental to developing tailored strategies aimed at improving pollen resilience to HS. During fertilization process, synergids are an essential part of the female gametophyte. These cells are involved in guiding the pollen tube to the embryo sac and facilitating the entry of the male gamete, and commit programmed cell death upon pollen tube arrival (Mendes et al., 2016). Ca^2+^ spikes were detected in the reception and recognition of the pollen tube by the synergid cells and ultimately upon pollen tube burst and delivery of the male gametes and upon synergid cell death (Ngo et al., 2014). Studies understanding how Ca^2+^ spikes relationship with HS in the context of fertilization process is crucial for developing effective strategies to mitigate the negative impacts of HS on seed production and ensuring sustainable practices in the face of climate change.

## HS effects in a crop of economic relevance, *Oryza sativa*

**Figure 3:**
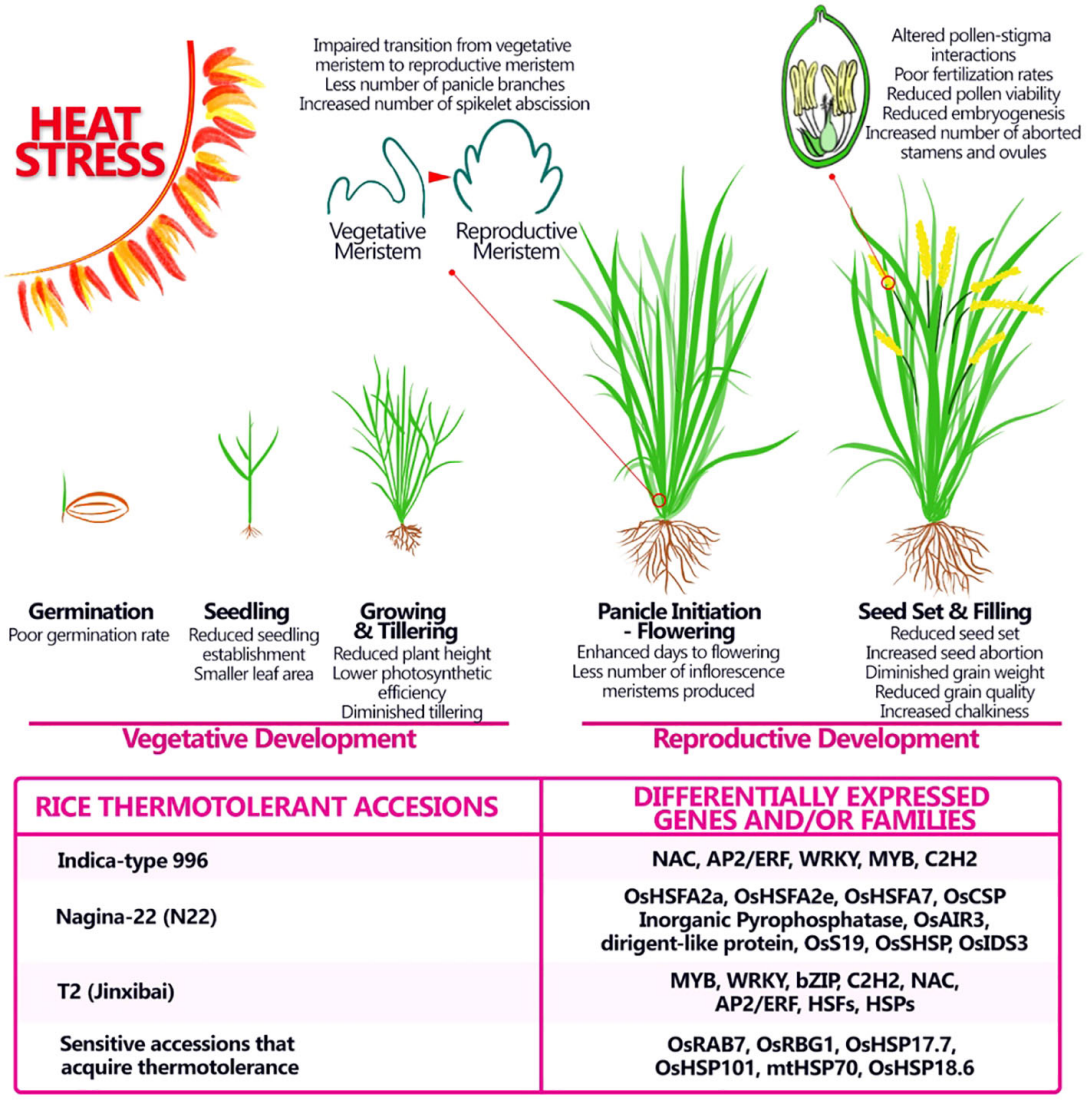
Adverse impact of heat stress on morphological and physiological aspects of rice at different stages of development and possible genes and/or families involved in thermotolerance. Heat stress affects rice productivity by affecting both vegetative and reproductive stages, still major effects of heat stress are seen during the reproductive stage and can provoke alterations in the number of panicles produced in the latest stages, modifying the starch content and therefore the quality of the grain. Some thermotolerant accessions were studied and specific gene or families involved in the thermotolerance were discovered.

Rice, like several other cereal species, shows large adaptive phenotypic plasticity enabling yield stability across environments. However, high temperatures beyond the critical threshold of rice growth can cause severe reductions in grain yield and quality, particularly from the heading stage to the grain-filling stage. Generally, the process of male and female gametophyte formation in *Oryza sativa* is similar to what was described for Arabidopsis, is mainly divided into three stages: meiotic division of the spore mother cell, mitotic stage of functional spore cells, and mature stage of gametophyte (Itoh et al., 2005). Research focusing on rice ovule and pollen development are limited for several reasons, the main one is the fact that the gametophytes are deeply embedded in the inflorescences also called panicles, because of their conic shape (Li et al., 2023). Rice panicle comprises the main axis, a branch from the branch meristem, and a spikelet from the spikelet meristem. The spikelet meristem forms the sterile organs, glumes and lemmas that enclose the florets which on in its turn contains all the reproductive and fertile organs that give rise to seeds (grains) (Itoh et al., 2005). The flowering phase in rice is highly sensitive to high temperatures. Two days of HS conditions resulted in an increase in the number of spikelets with non-viable pollen, meanwhile, four or more days of HS led to complete male sterility and several morphological defects on panicle development in heat-sensitive variety Nipponbare (**Figure 3**) (Endo et al., 2009, Li et al., 2023). To gain insight into the molecular mechanism of heat-induced male sterility, Endo and collaborators analyzed transcriptional alteration in the anther under high-temperature conditions using DNA microarray. The identified high temperature-repressed genes, such as YY1 and YY2 were expressed predominantly in the tapetum at the uninucleate microspore stage. Among them two genes involved in lipid metabolism, a plant-specific cytochrome P450 and a GDSL type ligase were identified, suggesting that the composition of lipid derivatives in the pollen might be altered in anthers exposed to high temperatures (Endo et al., 2009). Another study (Zhang et al., 2012) evaluated transcriptomic changes accompanying HS in reproductive tissues, at early stages of development (pre- and during meiosis) from the heat-tolerant cultivar Indica-type 996, which exhibits better anther dehiscent, pollen fertility rate and final seed yield than heat-sensitive cultivar Indica-type 4628 (Luo et al., 2005; Zhang et al., 2008). The predominant transcription factor gene families responsive to HS were HSF, NAC, AP2/ERF, WRKY, MYB, and C2H2, showing time-dependent gene expression pattern under short/middle-term HS (from 20 minutes to 8 hours). Furthermore, the promoter analysis of HS early up-regulated genes showed the important role of some specific motifs, such as HSE, GCC box, ABRE and CE3 in response to HS (Zhang et al., 2012, **Figure 3**). It is widely known that the HSE motif can be recognized and bound by HSF to respond to heat shock (Yamamoto et al., 2005), while other motifs are linked to ethylene, ABA and Ca^2+^ signaling, suggesting the existence of a complex crosstalk between several hormones and stimuli during heat shock (Zhang et al., 2012). Recently another thermotolerant rice accession was described, T2- Jinxibai, that after 45°C for 24h, exhibited high resistance to HS and the seedlings exhibited a survival rate of 90.93% after heat treatment. Sixty transcription factors were differentially expressed in the thermotolerant accession including the members of the AP2/ERF, NAC, HSF, WRKY, and C2H2 families as seen for the thermotolerant Indica-type 996 cultivar (He et al., 2023, **Figure 3**).

Very interestingly, Jagadish et al. (2008) describes the heat tolerant cultivar *Oryza sativa* indica Nagina 22 (N22), which after a 6-hour high temperature treatment, still maintains a 71% of spikelet fertility. This is a positive significant value if compared with sensitive (Moroberekan - 18% fertility) or intermediate (IR64 - 48% fertility) cultivars (Jagadish et al., 2010). Conducting a proteomic examination of the anthers in the heat-tolerant cultivar N22 unveiled the distinct expression patterns of 13 proteins. Among these, seven proteins demonstrated sequence similarities to potential cold shock protein (CSP), an inorganic pyrophosphatase, a serine protease (AIR3), a dirigent-like protein, a ribosomal protein (S19), a small heat shock protein (sHSP), and an iron deficiency protein (IDS3). These proteins are presumed contributors to the observed heightened heat tolerance in the cultivar. Particularly interesting are the stress-responsive cold and heat shock proteins identified (Jagadish et al., 2010, **Figure 3**), which will require further analysis to determine their role in HS tolerance. Consequently, it has been proposed that heat shock proteins contribute to higher tolerance to HS in rice. Supporting this hypothesis, Sailaja et al. (2015) reported that the expression of several heat shock expression factors (HSFS - OsHSFA2a, OsHSFA2e, and OsHSFA7) were highly upregulated in N22 plants when they were under heat treatment (42°C for 24 hours), with respect to the heat susceptible cultivar Vandana. Only two HSFS (OsHSFA2e and OsHSFA7) were upregulated under the same heat treatment in the susceptible cultivar Vandana, although the increase on the level of expression was minimal when compared with N22.

N22 cultivar also presented an improvement in the photosynthetic rate and chlorophyll fluorescence, and reduced transpiration rate under HS. These traits may help this cultivar to show better performance on HS conditions and hence superior yield (Vivitha et al., 2018). The N22 cultivar was also used in another study to depict the global transcriptional response to HS in reproductive tissues, specifically during anthesis (González -Schain et al., 2016). It has been well documented that anthesis in rice is the stage most sensitive to high temperatures (Prasad et al., 2006; Jagadish et al., 2007), during which many physiological processes occur in less than one hour. Indeed, it was shown that reproductive tissue responds quickly, already after 30 minutes, to adjust their transcriptome to prevent damage produced by high temperature (38°C). Proper expression of protective chaperons in anthers at anthesis is needed to overcome stress damage and to ensure fertilization (González -Schain et al., 2016).

Similar to Arabidopsis, in the case of rice, there are relatively few instances where the impact of temperature stress on female reproductive processes has been explored. However, a more extensive body of knowledge exists regarding the effects of HS on male reproductive functions.This is because pollen is easily accessible compared to ovules coupled with the notion that pollen exhibits greater sensitivity to HS than female reproductive organs in different crop plants (Hedhly et al., 2009; Wang et al., 2021). However, recent studies revealed varying degrees of sensitivity of the pistil, ovaries, ovules, and gametophyte to the HS depending on rice varieties and developmental stages (Wang et al., 2021; Shi et al., 2022). Shi and colleagues (Shi et al., 2022) demonstrated the heat sensitivity of the pistil showing that HSed pistil pollinated with non-stressed pollen resulted in a significant reduction in spikelet fertility in the sensitive IR64 cultivar at 40°C. On the contrary, no sensitivity was observed in the N22 variety, indicating tolerance of N22 to HS also during pistil development. Interestingly, a significant proportion of ovules of IR64 variety subjected to HS were characterized by a non-corrected differentiation of megaspore mother cell or by the degeneration of all four megaspore cells instead of three after meiosis. All those effects resulted in a lesser proportion of viable embryo sacs, such as mature embryo sacs lacking the egg cell or the central cell (Shi et al., 2022). In addition to the effects on gametophyte, a previous study reported the effects of heat on the tissues of the pistil. In particular, about half of the spikelets observed at the SEM microscope developed pistil hyperplasia, i.e., proliferated female organs or tissues, including multiple stigmata and/or ovaries, and differentiation of trichomes from ovary epidermis (Takeoka et al., 1991). HS reduces the capacity of rice grain to assimilate supplies, such as starch and proteins, additionally also shortens grain-filling stage duration, leading to the reduction of grain weight and a chalky-appearing grains, greatly damaging their market value (Kobata and Uemuki, 2004; Peng et al., 2004). Given that starch constitutes the primary component of grains, its deficiency is a key factor contributing to the reduction in grain weight under high temperatures. Consequently, transcriptomic studies have demonstrated that HS suppresses the expression of genes involved in starch biosynthesis while promoting the expression of enzymes responsible for starch consumption (Yamakawa et al., 2007). For example, heightened temperatures led to increased expression levels of several α-amylase genes, namely *Amy1A, Amy1C, Amy3A, Amy3D, and Amy3E*, along with an elevation in enzyme activity. In contrast, the expression of starch biosynthetic genes such as granule-bound starch synthase I *(GBSSI*) and a starch branching enzyme *(BEIIb)* was reduced (Yamakawa et al., 2007). Subsequent research confirmed that the expression of *Amy1A, Amy3C, and Amy3D* in the endosperm during seed ripening significantly contributes to the production of chalky grains in high-temperature conditions (Nakata et al., 2017). Furthermore, the downregulation of two key sucrose transporter genes, namely SUT1 and SUT2, under HS indicates a potential hindrance to the import of sucrose into the endosperm (Yamakawa and Hakata, 2010).Those results were supported by a parallel metabolomic analysis showing that sucrose and amino acids accumulated, and the level of sugar phosphates and organic acids decreased in HS-ripened caryopses (Yamakawa and Hakata, 2010). Thermotolerance in rice during both vegetative and reproductive growth without a yield penalty was recently identified by a natural quantitative trait locus (QTL), *TT2 -THERMOTOLERANCE 2.* TT2 encodes a Gγ subunit that codifies for a heterotrimeric GTP-binding proteins (G proteins), the thermotolerance was directly linked to the SCT1 (Sensing Ca^2+^ Transcription factor 1) - dependent alteration of wax biosynthesis. The calmodulin–SCT1 interaction was attenuated by reduced heat-triggered Ca^2+^ caused by disrupted TT2 (Kan et al., 2022).

Recently, the allele of the *TT1* gene coming from African rice (*O. glaberrima* - CG14), which encodes for a 26S proteasome α2 subunit protein, boost thermotolerance by enhancing the recycling and elimination of denatured ubiquitinated proteins consequence of HS. Remarkably, plants harbouring the *TT1*-CG14 allele greatly outperformed plants carrying the Asian rice (*TT1*- *O. sativa* spp. *japonica*) allele in grain per plant production after heat treatment (12h at 38°C/12h at 35°C for 5 days). Yield superiority conferred by the *TT1*-CG14 allele, was observed regardless if HS was applied during flowering or grain filling stages. These results validate the potential of the *TT1*-CG14 allele for breeding heat tolerant crops (Li et al., 2015).

Likewise, plants harbouring the TT3 QTL from CG14 presented higher survival rate at reproductive stage and improved grain yield after HS treatment (30 days at 38°C/34°C day/night) compared with plants carrying the Asian rice TT3-QTL (from *O. sativa* spp. *japonica*). TT3 quantitative trait loci contains the *TT3.1* and *TT3.2* genes, TT3.1 is a RING-type E3 ligase and TT3.2 is a chloroplast precursor protein (ubiquitinated by TT3.1). After HS, TT3.2 is accumulated in chloroplasts causing damages to the photosystem II complex, compromising the thylakoid stability. Consequently, the improved E3 ubiquitin ligase activity of TT3.1-CG14 ubiquitinating TT3.2 for its rapid vacuolar degradation, protect the thylakoids from HS, hence increasing the thermotolerance of the plants (Zhang et al., 2022).

El-Esawi and Alayafi (2019) demonstrated that the overexpression of OsRAB7 enhances not only HS tolerance but also increased grain yield. The RAB protein family is involved in multiple developmental processes and has been linked to tolerance to environmental stresses (reviewed in Tiwari et al., 2021). Transgenic plants overexpressing OsRAB7 presented an increment of nearly 40% in survival rate after a heat treatment (40°C day/32°C night, irrigated daily, for 10 days) with respect to the wild type individuals. Under HS conditions, both wild-type and overexpression lines presented diminishment in growth when compared to individuals growing in normal conditions. However, transgenic lines exhibited better growth performance when compared to the wild type under HS conditions. These findings suggest that the increased expression of OsRAB7 in transgenic rice plants positively influences their survival rate, growth, relative water content, and resilience against both drought and HSes. Given that OsRAB7 overexpression has been associated with improved salt tolerance in rice by enhancing stress signaling transduction through intracellular vesicle trafficking (Peng et al., 2014), it is highly likely that heightened HS tolerance is also achievable through enhanced intracellular vesicle trafficking (El-Esawi and Alayafi, 2019). The yield-related improved traits on the OsRAB7 overexpressing lines when compared with the wild type were: panicle length (+25%), number of spikelets per panicle (+11%), total number of spikelets per hill (+11%), number of filled grains per hill (+35%), filling rate (+21%), and total grain weight (+27%). It is important to notice that under normal conditions, the OsRAB7 overexpressing lines did not show any significant difference on yield traits compared to the wild type (El-Esawi and Alayafi, 2019). These data are extremely important because represent one of the few examples of reported transgenic lines that display a better yield performance under HS conditions. Indeed, most of the improved stress tolerance transgenic lines are focused on the survival rate of the plants but no data regarding the effect on yield is shown. Recently, Lo et al (2020) identified a novel *RICE BIG GRAIN 1 (RBG1)* gene that is involved in auxin homeostasis and enhances cell division. The overexpression of *RBG1* impacts several aspects of plant growth and development including a significant enhancement in the size of the panicle and seeds, when compared to wild-type plants. This positive yield effect, together with the fact that 31 members of the HSPs gene family resulted upregulated on *RBG1* overexpression lines when compared with wild type plants, led the researchers to evaluate the performance of these lines under several stress conditions including HS (4 days at 42°C). Notably, the RBG1 overexpression lines showed a higher survival rate after recovery (≈80%) than the wild-type plants (≈20%). However, if the positive effect on yield properties that confers the overexpression of RBG1 is still manifested under HS conditions remains unclear. Undoubtedly, OsRAB7 and OsRBG1 represent excellent candidates to be used in future breeding programs focused on dealing with climate change and raising the global temperature. Nonetheless, the performance under HS conditions of some other important yield-related traits such as plant height, number of panicles per plant, number of primary and secondary branches on the main panicle and thousand seeds weight, remain largely unexplored for these interesting transgenic lines (Lo et al., 2020).

Some natural allelic variations on *SLENDER GUY* 1 (*OsSLG1*), a cytosolic tRNA 2-thiolation protein, confer higher thermotolerance at both seedling and reproductive stages (Xu et al., 2020). The loss of function of *SLG1* reduced more than 80% the survival rate of the seedlings after heat treatment. Interestingly, *slg1* plants at reproductive stage after heat treatment (40°C for 5 days) showed reduced seed-setting rate generated by a large reduction on the number of pollen grains on the surface of the stigma and consequently of growing pollen-tubes. The authors proposed that the SLG1 tRNA-modification activity positively impacts the translation efficiency of the cell, thus modulating the concentration of HSPs and reducing the proportion of mis-folded proteins (Xu et al., 2020). In consequence, augmented translational efficiency and fidelity resulted critically beneficial to tolerate high temperature stress.

HSPs along with HSFs are the most important players in heat response transcriptional regulatory networks. In 2003, Katiyar-Agarwal and colleagues demonstrated that overexpressing Arabidopsis HSP101 in rice under high-salinity conditions improved yield by enhancing pollen tube viability. Other studies involving HSPs demonstrated that the survival rate for rice plants overexpressing the sHSP17.7 after a 2-hour at 50°C treatment increased with respect to the control (Murakami et al., 2004). In a clear example of “cross-tolerance”, rice lines overexpressing the sHSP17.7 protein were also capable of continuing to grow after a 6-day long drought period, while untransformed plants did not survive the treatment (Sato and Yokoya, 2008). The mitochondrial HS protein mtHSP70 is apparently involved in conferring heat tolerance resistance to rice protoplasts when overexpressed. Heat treatment (15 minutes at 48°C) on untransformed rice protoplasts resulted in around 27% survival rate, meanwhile protoplast overexpressing mtHSP70 presented a 60% survival rate (Qi et al., 2011, Li et al., 2023). These authors suggest that overexpression of mtHSP70 promotes increased HS tolerance on rice protoplasts by inhibiting programmed cell death triggered by high temperature through the maintenance of the mitochondrial membrane potential and preventing reactive oxygen species signal amplification (Qi et al., 2011). OsHSP18.6 is also capable of conferring enhanced HS tolerance when overexpressed. OsHSP18.6 overexpression lines displayed better root and shoot growth performance after a 3-week HS treatment (45°C/12h, 28°C/12h – Wang et al., 2015). Nonetheless, not all the HSPs increased HS tolerance when overexpressed. For example, overexpression of OsHsp17.0 and OsHsp23.7 did not improve HS tolerance with respect to wild type plants, but it enhanced salt stress and drought stress tolerances (Zou et al., 2012).

## Future perspectives

In response to HS, plants employ several mechanisms to maintain homeostasis and normal cellular functions. Understanding how these processes occur in the reproductive tissues of model species such as *Arabidopsis* is relevant for the establishment of a platform for advanced studies in crop species. Interestingly, HSPs are present not only in *Arabidopsis* but also in mammals, drosophila, yeast and so on. The studies of molecular mechanisms underlying tolerance of HS could also be important for understanding similar mechanisms in other species. The knowledge of the molecular mechanism due to HS derived from the characterization of putative thermotolerant related genes and pathways in model species might have a direct impact on other species, specifically in species with more economic relevance for humankind. Most of the consequences of climate change for agricultural production are expected to be negative, making the implementation of mitigation strategies much needed to adapt crops to these new conditions. Special attention is directed towards crops that are essential parts of the human caloric intake: rice, wheat, and maize (FAO, 2023).

A plant exhibiting HS tolerance can sustain its regular growth and uphold, or even boost, total yield production in elevated temperature conditions by modifying metabolic and/or structural characteristics (Wahid et al., 2007). The features associated with heat tolerance are influenced by multiple genes and are connected to the morphological and physiological adaptations in rice. However, there is a scarcity of information regarding stress avoidance and tolerance mechanisms specific to rice. Identification of the molecular basis of plant adaptation is fundamental to driving plant breeding into the development of novel varieties that can adapt to climate changes. In addition, genome editing tools could play a role in bolstering or hastening crop responses to climate change and/or biofortified crops to provide adequate nutritional quality to a growing population (Leisner, 2020). Rice, like several other cereal species, shows large adaptive phenotypic plasticity enabling yield stability across environments.

## Author contributions

FR: Conceptualization, Writing – original draft. GO-A: Conceptualization, Writing – original draft. MC: Writing – original draft. MM: Writing – original draft, Conceptualization, Writing – review & editing.
